# Plasma IGFBP-2 levels reveal heterogeneity in hepatic fat content in adults with excess visceral adiposity

**DOI:** 10.3389/fendo.2023.1222101

**Published:** 2023-10-04

**Authors:** Chloé Rauzier, Dominic J. Chartrand, Natalie Alméras, Isabelle Lemieux, Eric Larose, Patrick Mathieu, Philippe Pibarot, Benoît Lamarche, Caroline Rhéaume, Paul Poirier, Jean-Pierre Després, Frédéric Picard

**Affiliations:** ^1^ Centre de recherche de l’Institut universitaire de cardiologie et de pneumologie de Québec (IUCPQ) – Université Laval, Québec, QC, Canada; ^2^ Faculté de pharmacie, Université Laval, Québec, QC, Canada; ^3^ Département de kinésiologie, Faculté de médecine, Université Laval, Québec, QC, Canada; ^4^ Département de médecine, Faculté de médecine, Université Laval, Québec, QC, Canada; ^5^ Département de chirurgie, Faculté de médecine, Université Laval, Québec, QC, Canada; ^6^ Centre Nutrition, santé et société (NUTRISS), Institut sur la nutrition et les aliments fonctionnels (INAF), Université Laval, Québec, QC, Canada; ^7^ École de nutrition, Faculté des sciences de l’agriculture et de l’alimentation, Université Laval, Québec, QC, Canada; ^8^ Département de médecine familiale et de médecine d’urgence, Faculté de médecine, Université Laval, Québec, QC, Canada; ^9^ VITAM – Centre de recherche en santé durable, Québec, QC, Canada

**Keywords:** humans, insulin-like growth factor binding protein-2, visceral adiposity, liver fat, ectopic lipid deposition

## Abstract

**Lay summary:**

Obesity is frequently accompanied by a fatty liver. However, some individuals with high abdominal fat levels nevertheless have low levels of liver fat. Reasons for such discordant phenotypes are unclear. In this paper, we report that among asymptomatic individuals with high levels of visceral fat, low concentrations of IGFBP-2 in the circulation were associated with significantly higher hepatic fat content compared to those with high IGFBP-2 levels. We conclude that quantification of plasma IGFBP-2 concentrations may be useful to identify the early risk for liver fat accumulation in apparently healthy individuals without cardiovascular symptoms.

**Aim/hypothesis:**

Although excess visceral adiposity (VAT) is generally associated with increased liver fat (LF), recent evidence has revealed heterogeneity in LF content among adults with visceral obesity, potentially contributing to specific differences in cardiometabolic outcomes. Reasons for such discordant VAT-LF phenotypes are largely unknown. The present study aimed at assessing whether circulating levels of insulin growth-factor binding protein-2 (IGFBP-2) could be a useful biomarker in the identification of heterogenous and discordant VAT-LF phenotypes.

**Methods:**

A sample of 308 middle-aged Caucasian apparently healthy men and women without cardiovascular symptoms were studied for the present cross-sectional analyses. Fasting plasma glucose and lipid levels were assessed and an oral glucose tolerance test was performed. Hepatic fat fraction (HFF) was measured using magnetic resonance spectroscopy whereas VAT was assessed by magnetic resonance imaging. Plasma IGFBP-2 levels were quantified by ELISA. Participants were then classified on the basis of median VAT (81 mL) and IGFBP-2 levels (233 ng/mL).

**Results:**

Individuals with high levels of VAT were characterized by higher waist circumference, lower insulin sensitivity, as well as by higher plasma triglyceride and lower HDL-cholesterol levels. Plasma IGFBP-2 levels were inversely correlated with HFF (r = -0.39, *p* < 0.0001). Among men and women with high levels of VAT, those with low levels of IGFBP-2 had significantly higher HFF (7.5 ± 0.7%), compared to participants with high IGFBP-2 concentrations (3.2 ± 0.5%, *p* < 0.0001).

**Conclusion:**

In the presence of excess VAT, high IGFBP-2 concentrations are associated with low levels of LF. Although additional studies will be necessary to establish causality and further clarify the clinical implications of these observations, these findings are concordant with a novel function of IGFBP-2 in modulating susceptibility to non-alcoholic fatty liver disease (NAFLD) in the presence of visceral obesity.

## Introduction

Chronic positive energy balance results in excess body fat accumulation, not only in adipose tissue, but also in non-adipose tissue-containing organs, such as the liver. In particular, deposition of lipids in the abdominal region, whether as visceral adipose tissue (VAT) and/or liver fat (LF), has been established as an important factor in the development of cardiometabolic diseases (1). Non-alcoholic fatty liver disease (NAFLD) is diagnosed when hepatic fat fraction (HFF) is higher than 5% (2). Although LF typically develops proportionally as a consequence of dysfunctional subcutaneous adipose tissue also leading to VAT accumulation (3), recent data from large cardiometabolic imaging cohorts have revealed the existence of discordant VAT-LF phenotypes, with individuals having high levels of LF without the presence of excess VAT, or vice versa (4). These discordant phenotypes have also been shown to be associated with different clinical outcomes, with high VAT being mainly linked with cardiovascular disease events independently of LF levels, whereas a high LF content was found to be predictive of an increased risk of type 2 diabetes (T2D) independently of VAT (4). Reasons for such discordant VAT-LF phenotypes are largely unknown.

Insulin like-growth factor binding protein-2 (IGFBP-2) is a pleiotropic circulating factor involved in the modulation of energy metabolism (5). In mice and humans, low plasma levels of IGFBP-2 are associated with higher body weight, adiposity, insulin resistance, and the development of NAFLD (6–8). In this context, we tested the links between plasma IGFBP-2 and LF content associated with variation in VAT in a sample of asymptomatic adult volunteers who were involved in a cardiometabolic imaging study.

## Methods

The present study reports additional IGFBP-2 data quantified in a previously described cohort of 312 individuals, including details about recruitment of participants and approval by the local institutional review board (#20769) (9). Informed written consent was obtained from all participants prior to their inclusion in the study. Samples were taken and deposited in a biobank for future analyses. The biobank is administered by a management framework. The present specific study has received ethics approval from the local institutional review board (#21433).

Briefly, men and women between 35 and 66 years old were enrolled from the Québec metropolitan area for voluntary participation in the visceral obesity/ectopic fat and non-invasive markers of atherosclerosis: a cardiometabolic-cardiovascular imaging study (CMCV imaging study). Data used in the present analyses are the baseline characteristics of study participants. Data for plasma IGFBP-2 levels were missing for 4 individuals, resulting in a cohort of 308 men and women.

Inclusion criteria for baseline measurements were body mass index (BMI) < 40 kg/m^2^ and being nonsmoker for at least 12 months before enrollment. Participants presenting symptoms or being treated for cardiovascular disease (including cerebrovascular disease), dyslipidemia, hypertension, and diabetes were excluded. Were also excluded participants undergoing hormonal or corticosteroid therapy, presenting a cancer not in remission or with a contraindication for magnetic resonance imaging (MRI), were on any medication in the past 3 months, as well as women who were in postmenopause for less than 12 months before enrollment.

Weight and waist circumference were measured as described (10). Body composition was quantified by dual-energy X-ray absorptiometry on a Lunar Prodigy system (GE Healthcare, Madison, WI, USA) whereas levels of abdominal subcutaneous adipose tissue (SAT) and VAT were assessed by MRI as previously described (9). HFF was evaluated using magnetic resonance spectroscopy (MRS) by breath-hold single‐shot stimulated echo acquisition mode (STEAM) sequence exactly as described (9). Blood samples were collected from the antecubital vein after a 12-h overnight fast. Circulating triglyceride (TG), total cholesterol, HDL and LDL cholesterol levels were quantified as described (9). Oral glucose tolerance tests (OGTT) were performed in overnight-fasted individuals by ingestion of a 300 ml solution containing 75 g of glucose (9). Plasma glucose and insulin levels were quantified throughout the test, allowing calculation of the total area under their excursion curve (AUC) by the trapezoid method. The homeostasis model assessment of insulin resistance (HOMA-IR) was calculated as glucose x insulin/22.5. Plasma IGFBP-2 levels were quantified by ELISA (Alpco kit #22-BP2HU-E01, Salem, NH, USA) according to the manufacturer’s instructions. The detection limit was 0.2 ng/mL. The inter- and intra-assay coefficient of variability was < 10%.

To determine the variation in LF content related to both VAT and IGFBP-2, participants were classified according to their respective medians (above or below) in VAT (81.2 mL) and IGFBP-2 levels (233.4 ng/mL). Differences between these groups were assessed by two-way ANOVA with Bonferroni’s *post-hoc* test when appropriate. A mediation model was performed with SAS v9.4 (SAS Institute Inc., Cary, NC) to assess potential causal relationships between variations in HFF and the link between IGFBP-2 and VAT. Differences between groups were considered statistically different when *p* < 0.05. Data analyses were performed using GraphPad Prism v8.1.

## Results

Mean age of participants was 50.5 ± 8.6 years with a mean BMI of 26.0 ± 3.8 kg/m^2^. To test the hypothesis that IGFBP-2 could be related to discordant VAT/LF phenotypes, the study sample was classified on the basis of median VAT volume (81.2 mL) and median circulating IGFBP-2 levels (233.4 ng/mL). Individuals with high VAT were characterized by higher waist circumference and levels of insulin resistance as well as by higher triglyceride and lower HDL-cholesterol levels (**Table 1**). Individuals with high IGFBP-2 had lower BMI, more favorable indices of plasma glucose-insulin homeostasis, lower TG and higher HDL-cholesterol levels (**Table 1**).

**Table 1:** Anthropometric, biochemical and cardiometabolic characteristics of a sample of 308 adult participants classified on the basis of their median visceral adiposity (VAT) and median fasting plasma IGFBP-2 concentrations.

	Low VAT ≤ 81.2 mL		High VAT > 81.2 mL		*p*		
Variables	Low BP-2 ≤ 233.4 ng/mL n = 47	High BP-2 > 233.4 ng/mL n = 107	Low BP-2 ≤ 233.4 ng/mL n = 107	High BP-2 > 233.4 ng/mL n = 47	VAT (V)	BP-2 (B)	V x B
**Total sample N = 308 (% women)**	46.2	59.9	32.7	34.0			
Anthropometric variables							
BMI (kg/m^2^)	25.4 ± 3.4	23.0 ± 2.5	28.4 ± 3.3	26.7 ± 2.5	< 0.0001	< 0.0001	0.37
Waist circumference (cm)	85.8 ± 7.4	79.3 ± 7.2	98.0 ± 7.9	93.4 ± 8.0	< 0.0001	< 0.0001	0.30
Plasma glucose-insulin homeostasis							
Fasting glucose (mmol/L)	5.14 ± 0.47	4.99 ± 0.41	5.43 ± 0.65	5.30 ± 0.48	< 0.0001	0.03	0.88
AUC glucose (mmol/L×180 min)	1048 ± 223	997 ± 201	1249 ± 366	1084 ± 205	< 0.0001	0.002	0.10
Fasting insulin (pmol/L)	32.1 ± 12.1	25.6 ± 10.3	60.0 ± 30.1	39.5 ± 16.7	< 0.0001	< 0.0001	0.006
AUC insulin (pmol/L×180 min)	44025 ± 23268	37748 ± 22062	81578 ± 43259	46618 ± 24764	< 0.0001	< 0.0001	0.0004
HOMA-IR	1.02 ± 0.40	0.79 ± 0.35	2.05 ± 1.18	1.30 ± 0.60	< 0.0001	< 0.0001	0.006
HbA1c (%)	5.3 ± 2.7	5.4 ± 2.7	5.5 ± 4.0	5.4 ± 2.9	0.02	0.76	0.34
Plasma lipids							
Triglycerides (mmol/L)	0.94 ± 0.43	0.78 ± 0.37	1.45 ± 0.88	1.16 ± 0.52	< 0.0001	0.006	0.43
Cholesterol (mmol/L)	4.91 ± 0.83	4.87 ± 0.94	5.06 ± 0.87	5.27 ± 0.90	0.01	0.43	0.25
LDL cholesterol (mmol/L)	3.07 ± 0.83	2.89 ± 0.77	3.28 ± 0.80	3.44 ± 0.80	0.0001	0.92	0.08
HDL cholesterol (mmol/L)	1.68 ± 0.43	1.84 ± 0.48	1.36 ± 0.33	1.54 ± 0.42	< 0.0001	0.001	0.85
Cholesterol/HDL cholesterol	3.11 ± 0.96	2.76 ± 0.66	3.91 ± 1.10	3.63 ± 1.10	< 0.0001	0.008	0.77
LDL/HDL cholesterol	2.00 ± 0.85	1.67 ± 0.59	2.53 ± 0.81	2.40 ± 0.88	< 0.0001	0.02	0.30

Values are means ± SD. Data analyzed by 2 x 2 ANOVA with VAT and IGFBP-2 levels as main factors.

AUC, Area under the curve during glucose tolerance test; BMI, Body mass index; BP-2, IGFBP-2, Insulin-like growth factor binding protein-2; HbA1c, Glycated hemoglobin; HDL, High-density lipoprotein; HOMA-IR, Homeostatic model assessment of insulin resistance; LDL, Low-density lipoprotein; VAT, visceral adipose tissue

**Figure 1:**
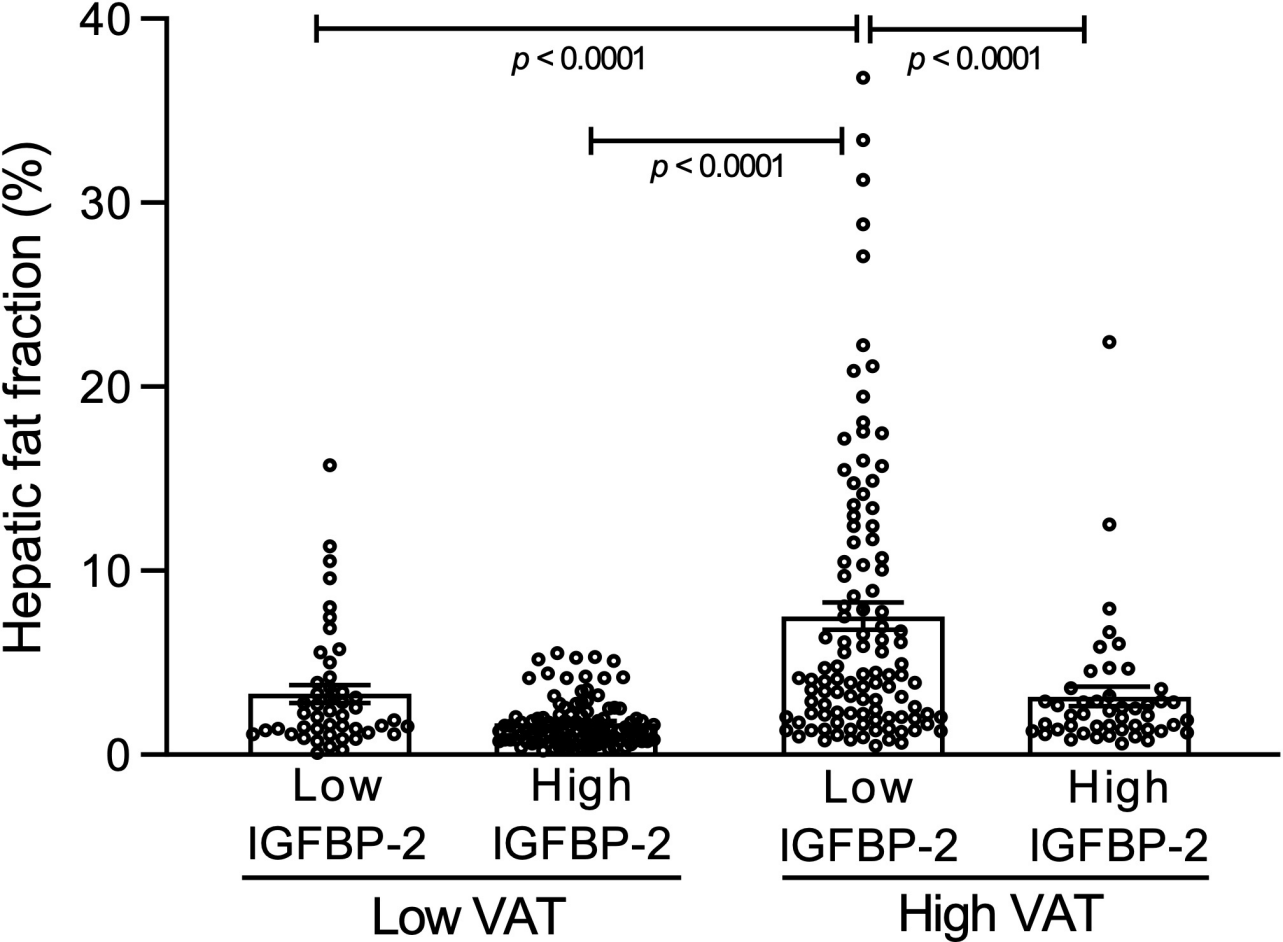
Hepatic fat fraction in a sample of 308 adult participants classified on the basis of their median visceral adipose tissue (VAT) (81.2 mL) and median fasting plasma IGFBP-2 concentrations (233.4 ng/mL). Each point represents one individual. Bars represent means ± SEM. *p* values were analyzed by *post-hoc* Bonferonni test performed after detecting a significant interaction (p=0.0246) upon 2 x 2 ANOVA with VAT (p<0.0001) and IGFBP-2 levels (p<0.0001) as main factors for hepatic fat fraction.

Plasma IGFBP-2 concentrations were inversely correlated with HFF (r = -0.39, *p* < 0.0001). Participants with low levels of VAT had an average HFF of 3.3 ± 0.5% and 1.7 ± 0.1% when IGFBP-2 levels were low or high, respectively (**Figure 1**). Strikingly, individuals with high VAT levels but low IGFBP-2 levels had a significantly higher HFF (7.5 ± 0.7%) compared to those with high IGFBP-2 concentrations (3.2 ± 0.5%, *p* < 0.0001, **Figure 1**). This statistically significant interaction between VAT and IGFBP-2 as main factors for HFF (*p* = 0.0246) was also observed for variables linked to insulin sensitivity (fasting insulin, AUC insulin, HOMA-IR) but not with waist circumference nor with plasma lipids (**Table 1**). Mediation analyzes revealed that the variation in HFF could be explained by up to 42.8% through the relationship between IGFBP-2 and VAT.

## Discussion

Results of this study show that in the presence of high levels of VAT, low circulating levels of IGFBP-2 are associated with the highest levels of LF. This novel finding is highly relevant because to the best of our knowledge no metabolic biomarker has yet been shown to convincingly discriminate levels of LF in the presence of visceral obesity (4). For instance, despite the fact that presence of excess VAT is associated with NAFLD at the population level, the two conditions are not always closely associated, resulting in different risks for various clinical outcomes (4). Although many studies have shown associations between IGFBP-2 and NAFLD in obesity (7, 11, 12), the present observations suggest that low plasma IGFBP-2 levels can indicate a higher risk for the development of NAFLD in asymptomatic, apparently healthy individuals with higher VAT. Moreover, in this subgroup, HFF levels were over the 5% threshold considered to indicate early NAFLD (2). In addition, in our study sample, median IGFBP-2 levels (233 ng/mL) were highly similar to the concentration of 220 ng/mL suggested as a cut-off value linked with the NCEP ATP III clinical criteria for the diagnosis of metabolic syndrome (13). However, additional studies conducted in larger and independent cohorts will be required to further validate these findings including thoroughly explore potential sexual dimorphism since it affects both IGFBP-2 and NAFLD (14, 15).

Our results also show robust statistical interactions between IGFBP-2 levels and VAT for indices of plasma glucose-insulin homeostasis, but not for lipid metabolism nor for waist circumference. It is interesting to note that a high LF content was recently shown to be more strongly associated with T2D than VAT (4), and that low IGFBP-2 was recently reported as one of the strongest biomarkers for T2D independently of age and sex (16). Given the established links between insulin resistance and the development of NAFLD (3), it is possible that IGFBP-2 contributes indirectly to a low LF phenotype by enhancing insulin sensitivity despite the presence of visceral obesity. This possibility is hinted by the 57.2% of variation in HFF that remain unexplained by VAT itself. Additional clinical studies using multivariate designs and direct experiments in models with modified IGFBP-2 expression will be needed to test this hypothesis.

## Data availability statement

The raw data supporting the conclusions of this article will be made available by the authors, without undue reservation.

## Ethics statement

The studies involving human participants were reviewed and approved by Comité d’éthique de l’IUCPQ. The patients/participants provided their written informed consent to participate in this study.

## Author contributions

EL, PM, PPi, BL, CRh, PPo, NA, J-PD, FP conceived and designed research project. CRa performed IGFBP-2 quantification. CRa, IL, and DJC analyzed data. CRa, NA, IL, J-PD, and FP interpreted the experimental results. CRa prepared the tables and figures. CRa and FP drafted manuscript. CRa, DJC, IL, NA, J-PD, and FP edited and revised the manuscript. All approved final version of the manuscript.
